# Cell-free DNA and next-generation sequencing in the service of personalized medicine for lung cancer

**DOI:** 10.18632/oncotarget.11717

**Published:** 2016-08-30

**Authors:** Catherine W. Bennett, Guy Berchem, Yeoun Jin Kim, Victoria El-Khoury

**Affiliations:** ^1^ Department of Oncology, Luxembourg Institute of Health, L-1526 Luxembourg, Luxembourg; ^2^ Centre Hospitalier de Luxembourg, L-1210 Luxembourg, Luxembourg

**Keywords:** lung cancer, cell-free DNA, next-generation sequencing, personalized medicine, biomarkers

## Abstract

Personalized medicine has emerged as the future of cancer care to ensure that patients receive individualized treatment specific to their needs. In order to provide such care, molecular techniques that enable oncologists to diagnose, treat, and monitor tumors are necessary. In the field of lung cancer, cell free DNA (cfDNA) shows great potential as a less invasive liquid biopsy technique, and next-generation sequencing (NGS) is a promising tool for analysis of tumor mutations. In this review, we outline the evolution of cfDNA and NGS and discuss the progress of using them in a clinical setting for patients with lung cancer. We also present an analysis of the role of cfDNA as a liquid biopsy technique and NGS as an analytical tool in studying *EGFR* and *MET*, two frequently mutated genes in lung cancer. Ultimately, we hope that using cfDNA and NGS for cancer diagnosis and treatment will become standard for patients with lung cancer and across the field of oncology.

## INTRODUCTION

Lung cancer is a leading cause of death worldwide, accounting for an estimated 1.6 million cancer deaths in 2013 [1]. In addition, lung cancer treatment is expensive and is the most expensive cancer type in Europe, costing 18.8 billion euros out of the 126 billion euros spent on cancer in 2009 and $12 billion in the United States in 2010 [2, 3]. As stated by Dr. Leonard Saltz at the 2015 annual meeting of the American Society of Clinical Oncology, new immunotherapies are even expected to drastically raise the cost of lung cancer treatments. Several types of lung cancer exist, including non-small cell lung cancer (NSCLC) and small cell lung cancer (SCLC). NSCLC accounts for 80-85% of lung cancer cases and can be further classified into three subtypes: squamous cell carcinoma, adenocarcinoma, and large cell carcinoma, while SCLC accounts for 15-20% of lung cancer cases [4]. Finally, tobacco smoking continues to be a significant risk factor in developing lung cancer, especially squamous cell carcinoma [5].

With the advent of advanced molecular techniques for diagnosing and treating diseases, personalized, or precision, medicine has emerged as a popular topic in the medical field. In personalized medicine, physicians use standard pathology information combined with molecular information to diagnose diseases and prescribe treatments catered to individual patient's needs [6]. Within the field of oncology, personalized medicine is at the forefront of cancer diagnosis and treatment. For example, cancer-specific biomarkers can be used to diagnose different cancer types [7]. In addition, mutational profiles of tumors can guide treatment options and help detect resistance to treatments, enabling physicians to evaluate therapeutic options quickly and effectively [8]. Lastly, proactive application of personalized medicine can be preventative by providing methods for detecting cancer risk factors and lessening them [9].

**Figure 1:**
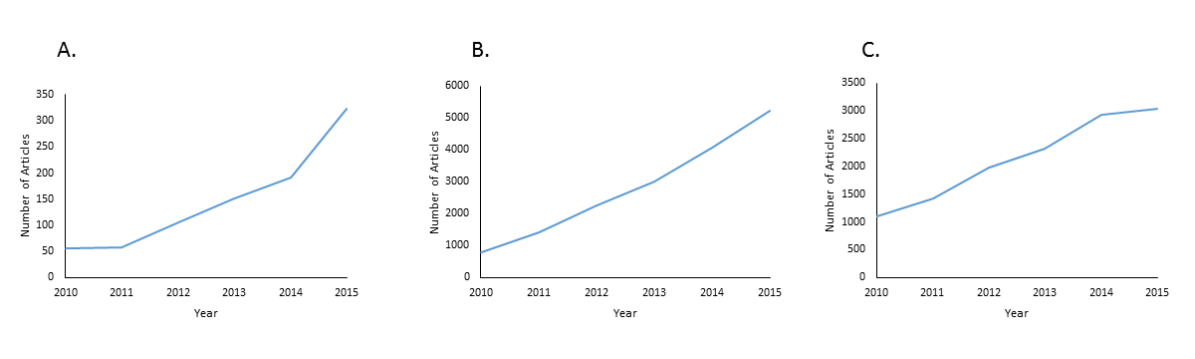
Rise of publications in cell-free DNA, next-generation sequencing, and personalized medicine **A.** Increase in publications regarding cell-free DNA from 2010 until 2015. Number of articles determined by Pubmed search of “cell-free DNA” OR “circulating free DNA.” **B.** Increase in publications regarding next-generation sequencing from 2010 until 2015. Number of articles determined by Pubmed search of “next-generation sequencing” OR “high-throughput sequencing.” **C.** Increase in publications regarding personalized medicine from 2010 until 2015. Number of articles determined by Pubmed search of “personalized medicine” OR “precision medicine.”

In the field of lung cancer research, personalized medicine has the potential to play a large role in diagnosing lung cancer and prescribing therapy. The ability to extract nucleic acids from tumor samples and detect mutations enables physicians to have access to large amounts of detailed genetic information. For instance, *EGFR* and *ALK* have been identified as key biomarkers in lung cancer, and molecular tests for *EGFR* and *ALK* have become common in lung cancer treatment [10]. If a patient tests positive for either of these mutations, lung cancer-specific tyrosine kinase inhibitors (TKIs) such as erlotinib, gefitinib, or crizotinib are prescribed [10, 11]. Two of the most important advancements in personalized medicine, especially in the field of lung cancer, include the use of circulating cell-free DNA (cfDNA) as a diagnostic and prognostic biomarker and next-generation sequencing (NGS) for mutational analysis of lung tumors. The importance of these tools is reflected in the increase in publications regarding cfDNA and NGS over the past five years (Figure 1). In this review, we present both of these innovations and their utility in diagnosing and treating lung cancer.

## CELL-FREE DNA

### cfDNA overview

Discovered in 1948, cell-free DNA (cfDNA) circulating in blood has emerged as a promising diagnostic tool for patients with cancer [12]. While the total amount of cfDNA in the plasma and serum of cancer patients varies from patient to patient, patients with cancer have higher average plasma and serum levels of cfDNA than patients without cancer [13–15]. In patients with lung cancer, plasma cfDNA levels are higher in later stage patients (mean: 38 ng/mL, 95% confidence interval (CI): 26-56 ng/mL) than in earlier stage patients (mean: 23 ng/mL, 95% CI: 18-30 ng/mL), and levels of plasma cfDNA over 100 ng/mL are more likely to be found in patients with SCLC *versus* NSCLC [15].

**Figure 2:**
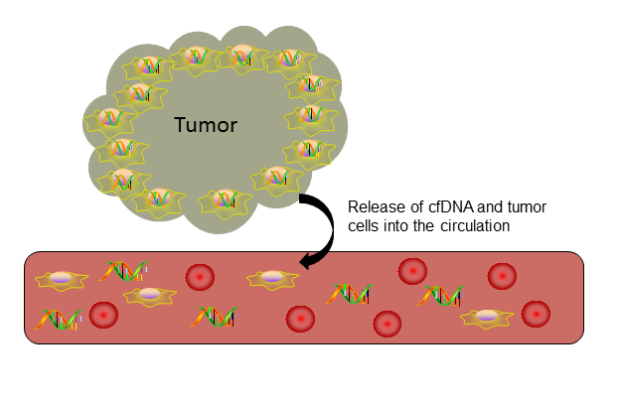
Release of cell-free DNA into circulation Cell-free DNA enters the bloodstream after apoptosis or necrosis or through active secretion by tumor cells.

Several hypotheses exist for the release of tumor DNA into the bloodstream, the most accepted being *via* apoptotic and necrotic tumor cells or by active DNA release by tumor cells (Figure 2) [14, 16]. According to the hypothesis that cfDNA is released during apoptosis or necrosis, as tumor cells divide, the apoptotic and necrotic tumor cells and DNA strands that are not phagocytosed enter the bloodstream as cfDNA [14]. Furthermore, in support of this hypothesis, cfDNA strands seen in the bloodstream are similar in length to the 180 base pair DNA strands that are characteristic of apoptosis [14, 16, 17]. More recent data suggest that cfDNA does not enter the bloodstream through apoptosis or necrosis but is actively released by cancer cells as a signaling molecule [18].

Indeed, cfDNA has been shown to have characteristics of a signaling molecule that induces metastasis of tumor cells. First, Garci-Olmo et al*.* showed that murine NIH-3T3 cells incubated with plasma from human colorectal cancer subjects positive for *KRAS* mutations developed *KRAS* mutations, and when these NIH-3T3 cells were injected into mice, tumors appeared, and human *KRAS* mutations were detected in mouse plasma [19]. Likewise, Trejo-Becerril et al. demonstrated that NIH-3T3 cells exposed to DNA from *KRAS* mutation-positive patient serum or cell supernatant developed a *KRAS* mutation over time, and when *KRAS*-positive cells plus the colon cancer carcinogen 1,2-dimethylhydrazine were injected into rats, the rats developed tumors with detectable *KRAS* mutations [20]. Taken together these studies suggest that a likely role for cfDNA is to act as a signaling molecule in tumor metastasis.

### Clinical potential of cfDNA in oncology

While the exact role of cfDNA remains elusive, it has clinical potential for detecting cancer, monitoring tumor mutations, and determining the effectiveness of treatment. In terms of cancer diagnosis, increased levels of cfDNA can be used as an indication of cancer across tumor types [21–24]. Not only can levels of cfDNA be used to distinguish cancer patients from non-cancer patients, but genomic analysis of cfDNA can also reveal known tumor mutations. To determine if cfDNA could be a reliable source for cancer mutation analysis, Lebofsky et al. compared the mutational status of plasma cfDNA samples to solid biopsy samples from 34 patients with metastatic cancer. In 27 of these patients, 28 out of 29 total mutations found in solid biopsy samples were also found in plasma cfDNA [25]. Similarly, when Janku et al. compared the mutational status of *BRAF, EGFR, KRAS,* and *PIK3CA* in plasma cfDNA samples to biopsy tissue samples, most mutations that were detected in the tumor biopsy samples were detected in plasma cfDNA samples: the concordant cases reached 91% for *BRAF* mutations, 99% for *EGFR* mutations, 83% for *KRAS* mutations, and 91% for *PIK3CA* mutations [26].

In addition to cfDNA found in plasma and serum, cfDNA in urine has shown promise as a biomarker for certain cancers. For example, in patients with non-muscle-invasive bladder cancer, high levels of cfDNA were found in urine samples in patients with progressive disease, including in samples from patients where levels of cfDNA were low in plasma [27]. Moreover, in a genomic analysis of urine cfDNA in patients with urothelial bladder cancer, there was a high rate of concordance between mutations found in urine cfDNA and tumor tissue. Importantly, circulating tumor DNA in urine had a sensitivity rate of 90% and permitted a better detection of genetic aberrations than urinary cellular DNA [28]. Taken together, these results indicate that urine cfDNA has a clinical utility for patients with cancer, especially as a minimally-invasive liquid biopsy technique.

Finally, cfDNA could be used to track response to treatment over time. In a study conducted to test this possibility, Frenel et al. tracked the mutational status of 39 late stage cancer patients over 11 months. At inclusion, 44 total mutations were detected in plasma cfDNA samples of 23 out of 39 patients, and at later time-points, four more plasma cfDNA samples harbored mutations [29]. Additionally, in plasma cfDNA from patients who were given targeted therapies, four patients exhibited a mutation allele frequency decrease, and two patients showed a mutation allele increase, indicating that mutations in cfDNA can be helpful in determining a patient's response to treatment [29]. Therefore, cfDNA is a viable clinical tool for tracking changes in tumor mutations and responses during treatment.

### cfDNA and lung cancer

For patients with lung cancer, cfDNA could be used as a diagnostic tool. In terms of diagnosis, plasma concentration of total cfDNA can serve as a biomarker for patients with lung cancer as patients with NSCLC have higher plasma cfDNA concentrations than healthy patients. Patients who were followed and did not relapse had lower plasma concentrations of cfDNA than patients who did relapse [21, 30]. These findings indicate that plasma cfDNA levels may aid in providing a diagnosis of NSCLC and in following tumor status over time. In addition, determining single nucleotide variants (SNVs) present in cfDNA samples could be useful for diagnosing lung cancer. In a pilot study using four patients with early-stage NSCLC, 16 SNVs were detected in cfDNA samples, and only one cfDNA sample bore 90% of the variants detected, while 22% and 33% of the variants found in the three other tumor samples were also found in cfDNA [31]. These results indicate that analysis of SNVs in cfDNA could have potential for diagnosing lung cancer in early-stage patients but that technological improvements are needed to increase the sensitivities of the assays. Finally, given that several inflammatory lung conditions increase plasma cfDNA levels, differentiating between patients with lung cancer and these conditions is an important diagnostic consideration. To that end, Szpechcinski et al. found that levels of plasma cfDNA were the highest in NSCLC patients and were significantly higher than plasma cfDNA levels in patients with inflammatory lung conditions (asthma, chronic obstructive pulmonary disease, and sarcoidosis) or healthy control patients [32].

While levels of cfDNA could serve as a diagnostic tool, the role of cfDNA levels as an indication of therapy effectiveness remains unclear. An early report suggested that NSCLC patients who progressed after chemotherapy had higher levels of plasma cfDNA than patients who did not progress and that patients who responded positively to chemotherapy had lower levels of serum cfDNA [33]. Additionally, in a study conducted with stage I NSCLC patients treated with stereotactic body radiotherapy, higher plasma cfDNA levels were found in patients who relapsed than in patients who had a positive response to therapy [34]. In contrast, a more recent report found that there is no correlation between levels of plasma cfDNA and response to platinum-based chemotherapy in patients with NSCLC [35]. Moreover, a report that examined the effects of several types of treatment (chemotherapy and tyrosine kinase inhibitors) on levels of plasma cfDNA found that there is no correlation between total plasma cfDNA levels and tumor prognosis in patients with late-stage NSCLC [36]. While these studies suggest that cfDNA levels may not be viable for monitoring response to treatment, they do not preclude the usefulness of analyzing tumor-specific mutations in cfDNA.

In order to determine if the emergence of somatic mutations indicating therapeutic resistance (resistance mutations) can be detected in cfDNA, Del Re et al. used droplet digital PCR (ddPCR) to analyze cfDNA samples from patients who had previously been treated with *EGFR*-TKIs. Resistance mutations were detected in 27/33 samples, including 11 samples with the T790M *EGFR* mutation, three samples with a mutation in *KRAS*, and 13 samples with both mutations [37]. In eight cases, tumor biopsies conducted after treatment were available, and there were 62.5% and 37.5% concordance rates for *EGFR* and *KRAS*, respectively [37]. Therefore, analysis of mutations in cfDNA, rather than levels of cfDNA, could provide insights into treatment effectiveness.

Finally, to evaluate the usefulness of cfDNA for the detection of epigenetic modifications in lung cancer, Lee et al. analyzed the methylation of *TMEFF2* in serum cfDNA samples from patients with NSCLC. They found *TMEFF2* methylation in 29/316 samples and no *TMEFF2* methylation in control samples, and when compared to corresponding solid tumor samples, three serum cfDNA samples matched tumor DNA samples [38]. Also, a study by Wang et al. found that increased *APC* and *RASSF1A* methylation levels in plasma cfDNA 24 hours post chemotherapy were correlated with a positive response to treatment in patients with advanced lung cancer [39]. In addition, in a study conducted to determine the methylation status of the *DCLK1* promoter in plasma cfDNA from lung cancer patients across stages, 49.2% of plasma cfDNA samples were methylated [40]. Notably in this study, 73.7% and 39.1% of SCLC and NSCLC samples respectively exhibited *DCLK1* plasma cfDNA methylation [40]. Taken with the results of Fournié et al. [15], these results indicate that cfDNA levels and methylation status could be used to aid in differentiating between SCLC and NSCLC diagnosis. Lastly, cfDNA methylation has the potential to differentiate lung cancer from other inflammatory lung diseases. To address this, Wielscher et al. found methylation levels of *HOXD10, PAX9, PTPRN2,* and *STAG3* to be higher in serum and plasma cfDNA samples from lung cancer patients than in patients with interstitial lung disease and chronic obstructive pulmonary disease [41]. In light of these results, the epigenetic status of plasma cfDNA is a promising biomarker for detecting lung cancer or a risk of lung cancer.

### Challenges presented by using cfDNA in routine clinical practice

cfDNA is undoubtedly a promising non-invasive biomarker, and analysis of cfDNA can support personalized medicine for cancer patients. Nevertheless, many issues must be addressed before it is widely accepted as a biomarker in clinical practice. First, technical challenges should be overcome, including (1) optimization of the protocols at the pre-analytical (sample collection, processing and storage), analytical (cfDNA isolation, quantification and mutational analysis) and post-analytical (data processing and interpretation) levels, (2) standardization of the operating procedures and (3) data validation in large multicenter studies [42]. Next, a deep and mature knowledge of cfDNA origin and clinical significance may facilitate the adoption of cfDNA as a liquid biopsy in clinics. Furthermore, a consistent detection of mutations in cfDNA, regardless of tumor stage, is an important step toward widespread physician approval of using cfDNA as a liquid biopsy. This can be achieved through combined improvements in the technical procedures for cfDNA isolation and an increase in the sensitivity of the genomic approaches for cfDNA analysis. Moreover, the validation of cfDNA assays for clinical diagnostics must meet the Clinical Laboratory Improvement Amendments (CLIA) requirements for the analytical and clinical performances of the test [43]. Also, if cfDNA analysis is going to be performed by next-generation sequencing, these sequencing platforms must be validated for clinical use [44]. Additionally, the NGS assay itself must be validated for cfDNA analysis, and the control used for validation should mimic the fragmented human cfDNA. Finally, standardized clinical guidelines need to be established, understood, and followed by the clinical team.

## NEXT-GENERATION SEQUENCING

### NGS overview

Next-generation sequencing (NGS) has emerged in the past decade as an efficient method for sequencing DNA and obtaining genetic information. Compared to the first generation sequencing method, Sanger sequencing, NGS is more efficient, more sensitive, and is becoming less expensive [45]. Indeed, the most obvious advantage of NGS over other techniques is the massively parallel sequencing feature. In a routine clinical setting, this property is important when several patients’ samples and different genomic regions have to be processed and data generated quickly. Another noteworthy advantage of NGS technology when compared to Sanger sequencing is its compatibility with low-quantity input DNA, as it is often the case with archived clinical samples. Moreover, its high sensitivity and reliability enable a more accurate detection of clinically important mutations. Finally, when multiple targets have to be analyzed, NGS technology considerably reduces the cost of screening compared to analysis with a lower throughput technology [44]. Despite the tremendous advantages of NGS, some challenges and considerations have to be taken into account for its complete implementation in a clinical setting. These issues are discussed in different sections of the present review.

The basic workflow of NGS is as follows: library preparation, library amplification, sequencing, and data analysis. While the workflow remains the same across platforms, the specific methods for library preparation and sequencing vary. Briefly, the Roche 454 and Ion Torrent^™^ platforms use emulsion PCR to collect single strands of DNA in each single bead, amplify DNA, and deposit amplified DNA on chips to be read by the sequencer [46, 47]. The Illumina platforms use a “cluster amplification” approach, whereby sample DNA is deposited on a flow cell and is amplified by bridge PCR [48]. To sequence, each company uses different technology to detect the addition of single nucleotides: luminescence (Roche), release of hydrogen ions (Ion Torrent^™^), and fluorescence (Illumina) [46–48]. In this way, whole genomes, exomes, transcriptomes, and targeted areas of the genome can be sequenced and mutations detected.

### NGS and lung cancer

In personalized medicine, NGS has several important applications and is useful in cancer diagnosis and treatment. In a study to determine if results from NGS are useful for detecting mutations in tumor tissues, Hagemann et al. analyzed the mutations revealed from NGS from the five most common cancer types and calculated a Shannon entropy level for each tumor type to determine if NGS revealed new information. High levels of Shannon entropy indicate analytic utility, while low levels indicate that a variable provides no new or useful information [49]. In this study, Shannon entropy levels for these cancer types from highest to lowest were colorectal cancer, high grade glioma, non-small cell lung cancer, pancreatic cancer, and sarcomas/soft tissue tumors [49]. These results suggest that for some major cancer types, including lung cancer, NGS has analytic utility and could provide useful information in cancer diagnosis without being redundant.

In cancer research, NGS can be used to detect mutations in tumors that might not have been detected with Sanger sequencing. For instance, in a study to determine mutations in lung and colon adenocarcinomas, NGS revealed three novel *KRAS* and *EGFR* mutations [50]. In addition, targeted NGS has been used to characterize similar mutations across different tumor types. In particular, Schwaederle et al. used NGS with solid tumor samples to determine if certain mutations were common to squamous tumor types. They found a set of genes more commonly mutated in either squamous (*TP53, PIK3CA, CCND1, CDKN2A, SOX2, NOTCH1,* and *FBXW7*) or non-squamous (*KRAS*) tumors [51].

In two comprehensive studies by The Cancer Genome Atlas (TCGA) Research Network, whole genome sequencing was used to determine commonly mutated genes in 178 lung tumor samples from patients with squamous cell carcinoma and 230 lung tumor samples from patients with adenocarcinoma. From the study with squamous cell carcinoma, notable results include a high rate of copy number alterations in *SOX2, PDGFRA, KIT, EGFR, FGFR1, WHSC1L1, CCND1,* and *CDKN2A*; a total of 228 non-silent and 360 exonic mutations; the detection of significantly mutated genes, including *TP53, CDKN2A, PTEN, PIK3CA, KEAP1, MLL2, HLA-A, NFE2L2, NOTCH1,* and *RB1*; overexpression of *SOX2* and *TP63*; inactivation of *CDKN2A* in 72% of cases; and *EGFR* amplifications in 7% of samples [52]. In addition, notable results from the study with adenocarcinoma include the identification of 18 genes that are commonly mutated in lung adenocarcinoma; determining somatic copy number alterations in *NKX2-1, TERT, MDM2, KRAS, EGFR, MET, CCNE1, CCND1, TERC, MECOM, CCND3,* and *CDKN2A*; the detection of *MET* exon 14 skipping; detecting mutations in *KRAS, EGFR, BRAF, ERBB2,* and *MET* that lead to activation of the receptor tyrosine kinase pathways; and determining frequently mutated activated molecular pathways in lung adenocarcinoma, including the RTK/RAS/RAF pathway, PI3K-mTOR pathway, and p53 pathway [53]. These results provide an overview of mutations in lung squamous cell carcinoma and adenocarcinoma and a baseline for future studies.

Additionally, in order to elucidate the genetic differences between lung neuroendocrine tumors, including SCLC, large-cell neuroendocrine carcinoma (LCNEC), typical carcinoid (TC), and atypical carcinoid (AC), 70 neuroendocrine tumor samples were sequenced using targeted NGS. Overall, low-grade tumors (AC and TC) had fewer mutations than high grade tumors (SCLC and LCNEC), and the following genes were associated with specific tumor types: *JAK3, NRAS, RB1,* and *VHL1* with SCLC; *FGFR2* with LCNEC; *KIT, PTEN, HNF1A,* and *SMO* with AC; and *SMAD4* with TC [54]. Likewise, in a study that examined the mutational status of tissue samples from patients with AC and TC using a targeted NGS panel, mutations (*BRAF, SMAD4, PIK3CA,* and *KRAS*) were only found in one out of 25 patients [55]. Taken together these two studies indicate that AC and TC tumors have distinct and fewer mutations than other pulmonary tumors with neuroendocrine features.

In other studies, NGS has been used to further characterize lung cancer mutations. First, Zhao et al. used their newly developed targeted NGS system to detect single nucleotide variants and indels in solid tumor samples of patients with lung cancer. One hundred and one mutations were found in a total of 168 genes, with *KRAS, TP53, EGFR, PIK3CA, BRAF, NRAS, JAK3, CTNNB1,* and *CKDN2A* being the most often mutated genes [56]. Moreover, 23 deletions, including deletions in *ARID4B* and *TP53* were detected [56]. Second, Iwakawa et al. performed whole exome sequencing and RNA sequencing on solid tumor samples from patients with SCLC. *TP53, RB1,* and *PTEN* were found to be highly mutated in both primary and metastatic SCLC tumors, and of these genes, *TP53* and *RB1* were found from tumors across stages, while *PTEN* was only found in tumors from stages II-IV [57].

Also, several gene fusions in lung cancer have been detected using NGS. For example, in NSCLC samples, simultaneous detection of *ALK*, *ROS1* and *RET* fusions and somatic mutations could be achieved in a very sensitive, specific, and tissue-sparing way using targeted NGS [58]. Similarly, a *FGFR3-TACC3* fusion was detected in a solid tumor sample from a patient with NSCLC for whom no other oncogenic mutations had been found using reverse transcription PCR, and this fusion was then detected in two more tumor samples that were thought to harbor no oncogenic mutations [59]. Of note, exome sequencing identified a germline mutation in *PARK2* that was associated with familial lung cancer [60]. In another study that considered the role of NGS in determining the inherited mutations of lung cancer, mutations were found in the *CLTCL1* and *PDE4DIP* genes in whole blood samples of family members with NSCLC [61].

Altogether, data from these studies demonstrate the utility of NGS in finding mutations in lung cancer and the ability to use NGS as a diagnostic tool for patients with lung cancer. Moreover, they suggest that NGS could be used to determine the genetic susceptibility for this disease.

### NGS and biopsy types

Given the potential for using NGS in the diagnosis of lung cancer, finding a biopsy that would provide reliable diagnostic information and would be less invasive to obtain than lung tumor tissue has emerged as a focus in lung cancer research [62]. Possible biopsies include fine needle aspirates (FNA) from tumor tissue, cfDNA from plasma and serum, bronchoalveolar lavage and pleural fluid, and cfDNA from urine. First, fine needle aspirates (FNA) have shown some success with detecting lung cancer mutations using NGS. When compared to paired formalin-fixed, paraffin-embedded (FFPE) tumor tissues, FNA from patients with lung adenocarcinoma had 99.9% overall sequence concordance, while only two single nucleotide polymorphisms from one patient were not detected in FNA tissue [63]. Also, when used to detect mutations in FNA samples from lung cancer metastatic specimens in lymph nodes, several mutations were found, including *EGFR* (34%), *KRAS* (8%), *BRAF* (3%), *PIK3C*A (9%), and *TP53* (36%) [64]. Therefore, NGS could be used with FNA tissue to detect lung cancer mutations in primary tumors and in metastatic sites.

Next, using NGS to find mutations in bodily fluids is possible due to the relative ease of collecting these samples. For instance, in a study to compare NGS results from plasma cfDNA and tumor samples using whole genome sequencing and targeted NGS, Xia et al. developed a plasma genomic abnormality (PGA) score to reflect mutational status and tumor burden. Patients with lung cancer had a higher plasma cfDNA concentration (4.9 ng per 400 μL, range 2.25-26.98 ng per 400 μL *versus* 2.32 ng per 400 μL, range 1.30-2.81 ng per 400 μL) and a higher PGA score (19.50, range 5.89-64.47 *versus* 9.28, range 7.38-11.08) than control patients, and targeted NGS revealed 14 point mutations in 12 genes in solid tumor tissue [65]. Also, to determine the mutations found in plasma and serum cfDNA samples of NSCLC patients, Paweletz et al. developed a targeted NGS panel for 11 oncogenes commonly associated with NSCLC. cfDNA samples were taken from patients with advanced NSCLC with known tumor genotypes. Mutations found in plasma cfDNA included *ALK, ROS1,* and *RET* rearrangements*, HER2* insertions*,* and *MET* amplification, and mutations in *BRAF* and *KRAS* [66]. The NGS results had sensitivities of 79% and 75% in the two subgroups of samples tested, and a specificity of 100% [66]. Moreover, two mutations that were not detected in tumor tissue were detected in cfDNA with the NGS assay (double deletion in exon 19 of *EGFR* in one patient and high levels of *MET* amplification in a different patient) and were confirmed with droplet digital PCR (ddPCR) and fluorescent *in situ* immunohistochemistry, respectively [66]. Furthermore, in a different study, there was a 76% concordance rate between mutations found in plasma cfDNA and mutations found in tumor samples from late-stage NSCLC patients, and additional mutations were found in cfDNA in several genes: *EGFR, KRAS, PIK3CA,* and *TP53* [67]. Lastly, another study by Vanni et al. compared mutations found in plasma cfDNA samples to those found in solid tumor samples from 12 NSCLC patients from various stages using targeted NGS. In the solid tumor samples (FFPE and snap frozen), all *EGFR* and *KRAS* mutations found with Sanger sequencing were also found using targeted NGS; however, in only one out of nine patients did the plasma cfDNA mutational profile match the solid tumor mutational profile [68].

It is noteworthy that the reported discrepancies may have occurred due to different assay sensitivities and to technical difficulties involved in handling low concentrations of DNA. Because levels of cfDNA are lower in early-stage patients, cfDNA samples in the Vanni et al. study might not have been concentrated enough to detect mutations. Moreover the NGS assay used in this study allowed the detection of mutations when the allele frequency was ≥ 5% [68], in contrast to the detection limit of 0.4% reported by Paweletz et al. [66]. Significant efforts have been undertaken to increase the sensitivity of cfDNA analysis [69–71], and these technological advances should be able to improve the current challenge associated with the accuracy of genotyping cfDNA.

Besides plasma, other bodily fluids have shown promise for mutational analysis. For example, mutations in bronchoalveolar lavage (BAL) and pleural fluids have been tested with NGS and yielded promising results. When NGS was used to test 48 BAL and pleural fluid samples for *EGFR* mutations, 81% of samples tested positive for *EGFR* mutations, compared to the 16% of samples that tested positive using Sanger sequencing [72].

Finally, given the relative ease of collecting urine samples, early data suggest that using NGS to determine mutations in urine cfDNA shows some promise for diagnosing lung cancer and monitoring response to treatment. For example, in TKI-pretreated patients with late stage NSCLC, the T790M mutation was found in 71% of urine cfDNA samples and in 75% of tissue samples, as assessed by the Trovagene quantitative PCR-NGS EGFR T790M assay and the Therascreen^®^ EGFR RGQ polymerase chain reaction test, respectively. Importantly, when tumor tissue was used as a reference, 93% of T790M-positive patients were also positive for this mutation in cfDNA from urine samples ≥ 90 mL. This percentage dropped to 72% when all urine volumes (from 10 to 100 mL) were considered (Gadgeel et al., poster presented at 2015 AACR-NCI-EORTC International Conference). Furthermore, using a similar approach to detect the T790M resistance mutation, another group found that the sensitivity of cfDNA for the detection of the T790M mutation in urine was 100% when using the mutational status of the tissue as a reference. Interestingly, the T790M mutation could be detected in urine samples three months earlier than progression appeared on clinical radiographic scans (Husain et al., poster presented at 2015 ASCO Annual Meeting). Taken together, these studies highlight the importance of urine cfDNA and NGS to analyze mutations in patients with NSCLC. However, since the use of urinary cfDNA to detect and monitor lung cancer is very recent in the field of oncology, caution should be exercised when raising conclusions from the studies mentioned above, which should be considered as preliminary. Further large-scale studies are needed to more specifically characterize the use of NGS with urine samples for lung cancer management.

### Technical comparison of NGS library preparation kits

Currently, Illumina, Life Technologies, and Roche 454 sequencing are the leading companies that dominate the NGS market. Both the Illumina and Ion Torrent^™^ (Life Technologies) platforms have several options for creating NGS libraries that are relevant in oncology (Table 1). First, hotspot panels enable targeted NGS for specific mutational cancer hotspots. In these panels, areas of genes that are often mutated in cancer are targeted and enriched using multiplex-PCR based library preparation [73]. The technology used for the Ion Ampliseq^™^ (Life Technologies) and Truseq^®^ Amplicon (Illumina) hotspot panels is based on this library preparation approach and enrichment. In the Illumina kits, there is also a prior targeted capture of the regions of interest by means of oligonucleotide probes. Illumina's TruSeq^®^ Amplicon Cancer Panel targets 48 genes with 212 amplicons in order to detect somatic mutations in FFPE tumor samples. Other TruSeq^®^ panels include the TruSeq^®^ Custom Amplicon v1.5 kit in which researchers can design custom panels with up to 1,536 amplicons per reaction with as little as 50 ng of input DNA. The TruSeq^®^ Custom Amplicon Low Input Library Prep kit is particularly interesting for low input and FFPE samples because it requires as few as 10 ng of DNA per sample.

**Table 1:** Comparison of NGS panels and library preparation kits

	Minimum DNA Input	Sample Types	Content and target	Target or Amplicon Size	Library Preparation Time	Cost
Illumina TruSeq® Amplicon Cancer Panel	150 ng 250 ng	High quality gDNA, FFPE	212 amplicons for 48 genes	170-190 bp	Fewer than 7 hours	11,769 euros ($13,428) for 96 samples
Illumina TruSeq® Custom Amplicon v1.5	50 ng	Fresh, frozen, or FFPE	Up to 1,536 amplicons (custom number of hotspots)	150, 175, 250, and 425 bp	10 hours	Dependent on number of amplicons
Illumina TruSeq® Custom Amplicon Low Input Library Prep Kit	10-50 ng (depending on FFPE DNA quality)	Low input samples FFPE	Up to 1,536 amplicons (custom number of hotspots)	150, 175, and 250 bp	6.5 hours	Dependent on number of amplicons
Illumina TruSight® Cancer Sequencing Panel	50 ng	gDNA (FFPE compatibility not supported)	~4,000 probes for 1,700 exons on 94 genes and 284 SNPs	Cumulative target region: 255 kb.Individual region size enriched: 350-650 bp	1.5 days	Dependent on number of targets
Illumina TruSight® Tumor 15	20 ng	FFPE	250 amplicons for 15 genes	~150-175 bp on average	7 hours	2,354 euros ($2,686) for 24 samples
Illumina TruSight® Tumor 26	30-300 ng (depending on FFPE DNA quality)	FFPE	174 amplicons for 26 genes	165-195 bp	Fewer than 7 hours	5,885 euros ($6,715) for 48 samples
Ion AmpliSeq™ Cancer Hotspot Panel v2 (with primer pool)	10 ng	FFPE and Fine Needle Aspirates (FNA) (among others)	207 amplicons for 50 genes	111-187 bp	3.5 hours	Ion AmpliSeq™ Cancer Hotspot Panel v2: 216 euros ($246) for 8 reactions Ion AmpliSeq™ Library kit 2.0: 860 euros ($980) for 8 reactions
Ion AmpliSeq™ Comprehensive Cancer Panel	40 ng	FFPE and Fine Needle Aspirates (FNA) (among others)	16,000 amplicons for more than 400 genes	125-175 bp	3.5 hours	860 euros ($980) for 8 reactions
Roche GS FLX Titanium Rapid Library Preparation Kit	500 ng	Double stranded DNA	N/A	3 kb, 8 kb, or 20 kb inserts	Not available	$1,192 for 12 library preparations

Information from data sheets, communications, and websites from Illumina, Ion Torrent™, and Roche.

In order to determine the relevance of using NGS to detect mutations in cancer patients in routine clinical practice, Wong et al. used the Illumina TruSeq^®^ Amplicon Cancer Panel to sequence tumors from 854 patients recruited in the multi-institutional Cancer 2015 cohort study [74]. Although the mutations found were successfully validated by the Agena MassARRAY^®^ assay, this study reports several limitations, including not being able to sequence a number of patients’ samples due to limited amount of starting material (less than 50 ng) and poor quality DNA samples that did not pass the sequencing quality-control filters [74]. These issues could be addressed through the use of panels or technologies that require lower input DNA and through the refinement and optimization of technical procedures, from tissue fixation to FFPE block storage. Given that the TruSeq^®^ Custom Amplicon Low Input Library Prep kit is new, little data exist on its relevance in a clinical setting; however, given that it only requires 10 ng of input DNA and that it is compatible with challenging samples like FFPE, it may be suitable for use with cfDNA samples in which DNA is fragmented and is found in limited quantities.

Other panels from Illumina include the TruSight^®^ panels that target whole exons and noncoding regions of DNA as opposed to hotspot regions [75]. In cancer genomics, the TruSight^®^ Cancer Sequencing Panel targets 94 genes that are thought to contribute to cancer and 284 single nucleotide polymorphisms (SNPs). In addition, the TruSight^®^ Tumor 15 targets relevant regions in 15 genes that are often mutated in solid tumors, while the TruSight^®^ Tumor 26, the first commercially available small actionable gene panel, is designed to assess low-frequency mutations in 26 genes involved in lung, gastric, colon, ovarian cancer and melanoma [75]. Both panels are compatible with FFPE samples. Importantly, the Illumina TruSight^®^ Tumor 26 panel has been clinically validated and is currently proposed as a clinical test by several institutes and laboratories [75].

With Ion Torrent^™^ technology, several options are on the market for library preparation and targeted NGS. For example, the Ion AmpliSeq^™^ Cancer Panel v1 that covers 739 hotspot mutations in 46 genes and Panel v2 that covers 2855 hotspot mutations in 50 genes are used with the Ion AmpliSeq^™^ Library Kit 2.0 to generate a multiplex PCR-based library starting with only 10 ng of DNA. Challenging samples such as FFPE tissues are compatible with these kits. The Ion AmpliSeq^™^ Cancer Hotspot Panel v2 has now been validated for clinical use in accordance with the Clinical Laboratory Improvement Amendments (CLIA) requirements [43, 75]. Given the low DNA input required for this panel and its possible use with fragmented DNA, it has the potential to be used with cfDNA. Accordingly, Life Technologies launched the Ion Torrent^™^ LiquidBiopsy^™^ Platform for simultaneous mutational analysis of cfDNA, circulating tumor cells, and white blood cells from a single blood sample (10 mL of blood) using the Ion AmpliSeq^™^ Cancer Hotspot Panel v2 for library construction and the Ion PGM system for sequencing. In addition to the Ion Torrent^™^ hotspot panels, Ion Torrent^™^ also produces the Ion AmpliSeq^™^ Comprehensive Cancer Panel that targets the exons of over 400 oncogenes and tumor suppressor genes. This panel is compatible with low integrity DNA such as FFPE but requires 40 ng of starting DNA material that may prevent its use with cfDNA.

Finally, the Roche 454 platform has limited library preparation kits. The GS FLX Titanium Library Preparation kit requires 500 ng of input DNA and can be used with 3 kb, 8 kb, or 20 kb inserts. Given the large amount of DNA required for this kit, it is not relevant for detecting mutations in samples where there is a limited quantity of DNA (FFPE, cfDNA).

In addition to the solutions offered by Illumina and Life Technologies for sequencing cfDNA that are discussed above, different companies propose kits specifically designed to optimize library preparation and enrichment from low input, fragmented cfDNA. As claimed by the manufacturers, this is achieved through improvements in library yield and quality, particularly a higher conversion rate of DNA to sequencing-competent adaptor-linked fragments and uniform GC-unbiased sequence coverage of the original DNA sample. Some of these kits are even compatible with an amplification-free workflow in order to reduce GC bias. Below are the most relevant examples of library preparation kits for cfDNA sequencing.

First, the GeneRead^™^ DNA library I Core Kit, GeneRead^™^ Adaptors, and the GeneRead^™^ DNA I Amp Kit from Qiagen can be used for DNA library preparation and subsequent sequencing on Illumina platforms, starting with 1 to 100 ng of cfDNA. Next, Bioo Scientific^®^ has developed NEXTflex^™^ Cell Free DNA-Seq Kit, a library preparation kit for use with cfDNA on Illumina platforms. This kit requires only 1 ng of cfDNA, and library preparation can be completed in only two hours. Additionally, Swift Biosciences^™^ offers different possibilities for constructing libraries compatible with Illumina sequencing platforms, from only 10 ng of cfDNA. For whole genome sequencing, the Accel-NGS^®^ 2S PCR-Free DNA Library Kit is recommended. For targeted NGS, the Accel-Amplicon^™^ 56G Oncology Panel covers hotspots of 56 cancer-related genes using 263 amplicons and the Accel-Amplicon^™^ EGFR Pathway Panel covers hotspots of *EGFR*, *NRAS*, *KRAS* and *BRAF* genes using 17 amplicons. Moreover, New England Biolabs offers kits for library construction from challenging samples that are compatible with Illumina sequencing. Their newly released NEBNext Ultra II DNA Library Prep Kitis an improved version of the original NEBNext Ultra Kit and is amenable to library construction from as little as 500 pg of input FFPE DNA. This kit has been successfully used for cfDNA mutational analysis (communication by Dr. Christopher Smith, Post-Doctoral Research Associate at the Cancer Research UK, Cambridge Institute, in October 2015 to New England Biolabs). In April 2016, New England Biolabs released the NEBNext Direct Cancer HotSpot Panel kit, which relies on the hybridization-based capture method. This kit enables DNA enrichment from 190 targets in 50 cancer-related genes starting with as few as 10 ng of DNA and is suitable for use with cfDNA and FFPE samples. Furthermore, the ThruPLEX^™^ Plasma-seq kit from Rubicon Genomics enables library preparation from 1 to 30 ng of cfDNA in two hours. The ThruPLEX^™^ Plasma-seq kit can be used with Illumina platforms for whole genome sequencing or for target enrichment workflows. Finally, the KAPA Hyper Prep Kit from KAPA Biosystems provides a library construction protocol that is compatible with Illumina sequencing and is suitable for FFPE samples and cfDNA with as few as 1 ng of input DNA. This kit can be used for different NGS applications, including whole genome and target enrichment sequencing.

### Comparison of NGS platforms

Several studies have been conducted to compare NGS platforms. First, Quail et al. compared the Ion Torrent^™^ PGM, Illumina MiSeq, Illumina HiSeq, Illumina GAIIx and PacBio^®^ (Pacific Biosciences) platforms using microbial genomes covering different GC contents. Of note, this study shows the error rates between platforms (< 0.4% for Illumina, 1.78% for Ion Torrent^™^, and 13% for Pacific Biosciences) and the poor performance of the Ion Torrent^™^ PGM in sequencing the extremely GC-poor genome of *Plasmodium falciparum*, resulting in 30% of the genome being uncovered [76]. Moreover, differences were observed in the rates of correct SNP calls (68-76% for Illumina, 82% for Ion Torrent^™^, and 71% for Pacific Biosciences) and in the rates of false SNP calls [76]. Importantly, the Ion Torrent^™^ PGM generated more false positive SNP calls than the Illumina platforms. These results are in concordance with the previously reported higher base call accuracy of Illumina platforms when compared to the Ion Torrent^™^ PGM (Phred quality score (Q) > 30 for Illumina and Q20 for the Ion Torrent^™^ PGM, corresponding to an incorrect base call probability of 1/1000 and 1/100, respectively) [76, 77]. When compared to the Illumina and Ion Torrent^™^ platforms, the PacBio^®^ platform has high error rates (< Q10), generates reads at lower throughput, and is more expensive [76, 77]. Moreover, when comparing the DNA requirements of each platform for standard library preparation, Illumina and Ion Torrent^™^ surpass PacBio^®^ given that far less DNA is needed with the first two platforms [76]. These features may hamper the use of PacBio^®^ for applications where only limiting amounts of DNA are available such as cfDNA sequencing. By virtue of its very long reads (~ 1,500 bp for the first generation of PacBio^®^ RS system and > 10 kb for the PacBio^®^ RS II) PacBio^®^ sequencing is not suited for the fragmented cfDNA analysis but rather for *de novo* genome assembly [77]. Despite differences in data generated, the Ion Torrent^™^ PGM and Illumina platforms were comparable in terms of reliability of results when sequencing GC-rich or GC-balanced genomes such as the human genome [76].

Next, in a study that compared the Roche 454 and Ion Torrent^™^ PGM platforms as well as the mutation-specific platforms, Cobas^®^ z 480 analyzer (Roche) and Rotor-Gene Q (Qiagen), Hinrichs et al. looked for *KRAS* and *EGFR* mutations in 25 FFPE samples from lung cancer patients. In 14 out of 25 samples, *KRAS* mutations that had been previously detected by Sanger sequencing combined with high-resolution melting (HRM) were detected by the Cobas^®^, Rotor-Gene, and Ion Torrent^™^ platforms [78]. In 10 out of these 14 samples, the Roche 454 could also detect the relevant mutations. The four other “mutated” samples and one “unmutated” sample could not be analyzed with Roche 454 because of technical sequencing issues (generation of unspecific PCR products), while all of the clinically relevant *EGFR* mutations that had been detected with HRM and Sanger sequencing were detected with all four platforms [78]. Notably, the Roche 454 generated 53,000 high quality reads (ca. 360 reads/amplicon), while the Ion Torrent^™^ PGM generated 4,000,000 high quality reads (ca. 1,500 reads/amplicon) [78]. This study demonstrates that, compared to Roche 454, NGS sequencing on the Ion Torrent^™^ PGM platform is more informative and faster.

Finally, Li et al. compared mutations found in inherited cardiac disease patients on the Illumina MiSeq and the Ion Torrent^™^ PGM platforms. In terms of target enrichment, 98.8% of the target region was covered at least once with the Illumina MiSeq platform, and 98.0% of the target region was covered with the Ion Torrent^™^ PGM platform [79]. Moreover, both platforms had greater than 200 reads for every protein-coding region of each gene [79]. In addition, the Illumina MiSeq and Ion Torrent^™^ PGM sequenced 97.9% and 96.8% of the target respectively, with a variant calling sensitivity of 100% for the MiSeq and 99.1% for the PGM [79]. The Illumina MiSeq required one sequencing run that cost $959 ($64 per specimen), while the Ion Torrent^™^ PGM required three sequencing runs that cost $686 each ($137 per specimen) [79]. Furthermore, despite the cheaper cost of the Ion Torrent^™^ PGM per run and its faster run time (3x3 hours) when compared to Illumina MiSeq (27 hours), the increased hands-on time and technical complexity of the Ion Torrent^™^ PGM platform resulted in a higher cost per sample than the Illumina MiSeq [79]. Given that the results obtained by each platform were comparable, the differences between the two platforms are the cost and the total amount of time required per run.

When choosing a platform to use in the clinic, several factors are important to consider, including the price of the system, the price per specimen, the sensitivity of the system, the run time required, and the relative ease of data analysis and interpretation. In terms of cost, in 2013, the Ion Torrent^™^ PGM cost $75 K, the Illumina MiSeq system cost $125 K, and the Illumina HiSeq 2000 system cost $654 K [76, 79]. However, the Illumina platforms cost less per million bases ($0.03 to $0.04 with the Illumina HiSeq 2500 *versus* $0.1 with the Ion Torrent^™^ PGM) [77] and per specimen ($64 per specimen for the Illumina MiSeq *versus* $137 per specimen for the Ion Torrent^™^ PGM) [76, 79]. Given the studies presented above, despite some differences, the sensitivity of these systems and the amount of DNA required are relatively comparable (10 ng for low input kits for potential use with cfDNA, 50-250 ng DNA for other kits), so decisions need to be made based upon cost and time required to run. However, if cfDNA analysis is to be performed with NGS, one additional point to consider when deciding whether to purchase one platform *versus* another platform is the compatibility of the library preparation kit with the platform. Next, in terms of the run time, in general, the Illumina platforms take longer to run than the Ion Torrent^™^ platforms (27 hours for the MiSeq and 11 days for the HiSeq 2000 *versus* two to four hours for the Ion Torrent^™^ PGM) [76, 79]. Finally, in terms of data analysis, Ion Torrent^™^ Suite software is used for analysing data generated on Ion systems. The Ion Reporter software facilitates data analysis, annotation, and visualization. For Illumina platforms, variant calling is performed with the MiSeq Reporter software that uses an alignment algorithm called the Burrows-Wheeler Aligner (BWA).

### NGS compared to other detection methods

As NGS becomes more prevalent in molecular diagnostics, comparing results obtained by NGS to results obtained by other techniques is vital for validating the use of NGS in a clinical setting. Thus, when targeted NGS results were compared to results obtained by real-time PCR for tumor samples from patients with NSCLC, the two methods had high concordance rates for the three genes tested: 96.3% for *EGFR*, 98.7% for *KRAS*, and 100% for *BRAF* [80]. In addition, targeted NGS identified eight *EGFR* indels and SNVs that were not detected by the real-time PCR method used [80]. Moreover, when results obtained from targeted NGS were compared to results obtained by immunohistochemistry (IHC) for tumor samples from NSCLC patients with *EGFR* mutations, the fair sensitivity of the mutant-specific antibodies (58.4%) did not favor the replacement of DNA sequencing by IHC for the detection of *EGFR* mutations. However, it is noteworthy that the specificity of IHC using mutated EGFR antibodies is excellent (98.0%) [81]. Finally, in a study to compare hybrid capture-based NGS with mass spectrometry genotyping and fluorescence *in situ* hybridization (FISH), NGS revealed actionable genomic alterations in 65% of solid lung tumors that were classified as negative by the other non-NGS methods [82]. Based on these studies, NGS is a superior method for detecting targetable mutations in lung tumors and would provide more sensitivity in lung cancer diagnosis.

## GENETIC ANALYSIS

In the following section, selected studies that sought to characterize *EGFR* and *MET*, two frequently mutated genes occurring in lung cancer, using NGS or PCR-based techniques with cfDNA and solid tumor samples are presented, and results are summarized in Table 2a, 2b, 2c.

**Table 2a:** Results from selected mutational analysis studies

Reference	Cancer Type	Tissue Type	Methods	Genes/ Mutations	Results
[89]	NSCLC	Plasma cfDNA and tumor tissue	Therascreen® Real-Time PCR and peptide nucleic acid (PNA) clamp Real-Time PCR	EGFR exon 19 del, L858R	Therascreen® RT-PCR: 65.4% sensitivity (sens.), 100% specificity (spec.); 55% detection for exon 19 del. in plasma cfDNA compared to tissue, 100% detection for L858R in plasma cfDNA compared to tissue PNA clamp: 61.5% sens., 100% spec.; 50% detection for exon 19 del in plasma cfDNA compared to tissue, 100% detection for L858R in plasma cfDNA compared to tissue
[90]	NSCLC	Plasma and serum cfDNA and tumor tissue	PNA clamp Real-Time PCR (Taqman)	*EGFR* exon 19 del, L858R	PNA clamp (Taqman): 78% overall concordance rate between plasma/serum cfDNA and tissue, 83.9% exon 19 del concordance rate, 70.7% L858R concordance rate Overall survival (OS): Median OS was shorter in patients with L858R than exon 19 del in cfDNA (13.7 months, 95% CI 7.1-17.7, versus 30.0 months, 95% CI 19.3-37.7)
[91]	NSCLC	Serum cfDNA and tumor tissue	Amplification Refractory Mutation System (ARMS) and Scorpion real-time PCR	*EGFR*	NGS: 23.7% detection rate in cfDNA, 61.5% detection rate in tumor tissue; 56.9% false negative rate for cfDNA Progression Free Survival (PFS): For patients positive for EGFR mutations, those who received gefitinib had longer PFS than those who received carboplatin-paclitaxel.
[93]	NSCLC	Plasma cfDNA and tumor tissue	ARMS Real-Time PCR	*EGFR*	Overall sensitivity in cfDNA: 17.2% Higher sensitivity in later-stage patients: 1.6% in stage IA, 7.9% in stage IB, 11.1% in stage IIA, 20.0% in stage IIB, and 33.3% in stage IIIA Higher sensitivity with lower levels of tumor differentiation: 0%, 15.7% and 36.8% in highly, moderately and poorly differentiated tumors, respectively. Positive ratios of plasma cfDNA compared to tumor tissue: Exon 19 del: 22.5%, L858R: 7.0%, L861Q: 75.0%, exon 20 insertions: 14.3%; T790M, G719X, S7681: 0%
[94]	NSCLC	Plasma cfDNA and tumor tissue	Droplet digital PCR (ddPCR) and ARMS	*EGFR* exon 19 del, L858R	ddPCR of cfDNA compared to ARMS tumor analysis: Exon 19 del: 81.8% sens., 98.4% spec., 94.2% concordance L858R: 80.0% sens., 95.8% spec., 93.0% concordance
[95]	NSCLC	Plasma cfDNA and tumor tissue	ddPCR	*EGFR* exon 19 del, L858R, T790M	Exon 19 del: 76.5% sens., 100% spec., 86.2% concordance with tumor tissue before treatment L858R: 70.8% sens., 100% spec., 87.9% concordance with tumor tissue before treatment Response to TKI: 40 patients with either L858R or exon 19 del in cfDNA at baseline showed a decrease in mutant levels after treatment. The T790M mut. was detected in 8 patients 2-12 months before progression was detected radiographically and in 6 patients at progression.
[96]	NSCLC	Plasma cfDNA and tumor tissue	ARMS and combination of mutant enriched PCR (me-PCR) and denaturing high performance liquid chromatography (DHPLC)	*EGFR* exon 19 del, L858R	Me-PCR and DHPLC: 77.3% sens., 89.6% spec., 85.1% concordance between cfDNA (me-PCR and DHPLC) and tissue (ARMS) Response to TKI: In tumor tissue: Objective response rate (ORR) of 69.4% for patients with *EGFR* mutations; ORR of 13.0% for patients without EGFR mutations In plasma cfDNA: ORR of 64.5% for patients with *EGFR* mutations; ORR of 28.6% for patients without *EGFR* mut.
[97]	NSCLC	Plasma cfDNA and tumor tissue	ddPCR and NGS	*EGFR*	ddPCR: 74% concordance rate between cfDNA and tissue Survival: Longer PFS and OS for patients with *EGFR* mutations in cfDNA and tumor samples versus *EGFR* mutations only in tumor (Median: PFS: 12.6 months versus 6.7 months; OS: 35.6 months versus 23.8 months) ddPCR and NGS: Limit of quantification: 0.04% for ddPCR, 5% for NGS; NGS had 89% sens. and 100% spec.
[98]	NSCLC	Plasma cfDNA and tumor tissue	Digital PCR	*EGFR* T790M	Activating Tumor Mutations: 88.2% of tumor samples had *EGFR* mutations; 58.8% of plasma cfDNA samples had *EGFR* mutations. Resistance Mutations: T790M mutation in plasma cfDNA detected in patients after receiving EGFR-TKIs; 81.8% sensitivity, 85.7% specificity, 83.3% concordance between plasma cfDNA and tumor tissue

**Table 2b:** Results from selected mutational analysis studies

Reference	Cancer Type	Tissue Type	Methods	Genes/ Mutations	Results
[99]	NSCLC	Plasma cfDNA and tumor tissue	Plasma ddPCR and tumor genotyping	*EGFR* exon 19 del, L858R, T790M and KRAS mutations	ddPCR of cfDNA compared to tumor genotyping: *EGFR* Exon 19 deletion: 100% positive predictive value (PPV) and 82% sensitivity *EGFR* L858R: 100% PPV and 74% sensitivity *EGFR* T790M: 79% PPV and 77% sensitivity *KRAS* G12X: 100% PPV and 64% sensitivity
[100]	NSCLC	Plasma cfDNA and tumor tissue	**Cobas**® EGFR Mutation Test, Therascreen® EGFR Mutation Test, ddPCR and BEAMing dPCR	*EGFR* exon 19 del, L858R and T790M	**In a first set of 38 plasma samples:** **Cobas**® Test: 86% and 90% sens., 100% spec., 89% and 97% concordance with tumor tissue for *EGFR* exon 19 del and L858R, resp. Therascreen® Test: 82% and 78% sens., 100% spec., 87% and 95% concordance with tumor tissue for *EGFR* exon 19 del and L858R, resp. ddPCR: 90% sens., 100% spec., 97% concordance with tumor tissue for *EGFR* L858R BEAMing dPCR: 93% and 100% sens., 100% and 93% spec., 95% concordance with tumor tissue for *EGFR* exon 19 del and L858R, resp. **In a second set of 72 plasma samples:** **Cobas**® Test: 73% sens., 67% spec. for *EGFR* T790M BEAMing dPCR: 81% sens., 58% spec. for *EGFR* T790M
[101]	NSCLC with *EGFR* exon 19 del or L858R and acquired *EGFR*-TKI resistance, selected for AZD9291 treatment	Plasma cfDNA	BEAMing dPCR	*EGFR* T790M	Outcomes on AZD9291: If cfDNA *EGFR* T790M-positive, ORR of 63% and median PFS of 9.7 months If tumor *EGFR* T790M-positive, ORR of 62% and median PFS of 9.7 months Conclusions: If cfDNA is T790M-positive, no need for tumor genotyping. However, if cfDNA is T790M-negative, tumor genotyping is warranted
[102]	NSCLC with acquired resistance to AZD9291 treatment	Plasma cfDNA	NGS and ddPCR	*EGFR* exon 19 del, L858R, T790M and C797S	Upon AZD9291 treatment, different resistance phenotypes can emerge from *EGFR* T790M-positive patients : Acquisition of *EGFR* C797S, maintenance of T790M positivity without C797S or loss of *EGFR* T790M. Conclusions: Several mechanisms result in the emergence of resistance to AZD9291. Therapies that overcome resistance due to *EGFR* C797S mutation are needed.
[103]	NSCLC	Tumor tissue	Targeted NGS and Real-Time PCR	*EGFR* T790M and other EGFR and non-EGFR mutations	NGS: T790M detected in 60.0% of patients (all patients previously treated with EGFR-TKIs); Other mutations detected: *TP53* P72R (86.7%), *KDR* Q472H (33.3%), and *KIT* M541L (13.3%); NGS is able to detect T790M mutation better than real-time PCR
[104]	NSCLC	Tumor tissue	NGS deep sequencing	*EGFR*	NGS: 24.6% of samples had compound mutations; 66.7% of compound mutations had an atypical mutation with *EGFR*-TKI sensitizing mutation Survival: Shorter OS for patients with compound mutations (72.8 months) versus patients without compound mutations (83.7 months) Co-occurring mutations: Patients with compound mutations are more likely to have co-occurring mutations in other genes than patients with simple mutations
[105]	NSCLC	Plasma cfDNA, malignant pleural effusion (MPE), and tumor tissue	ARMS for all samples, Sanger sequencing and immunohistochemistry (IHC) for MPE cell block and tumor tissue samples	*EGFR*	ARMS: In MPE cell block samples compared to tissue: 81.8% sens., 80.0% spec., 81% concordance In MPE supernatant compared to tissue: 63.6% sens., 100% spec., 81% concordance In plasma cfDNA compared to tissue: 67.5% sens., 100% spec., 84.9% concordance In MPE supernatant compared to MPE cell block: 69.2% sens., 100% spec., 85.2% concordance Sanger sequencing compared to ARMS: In tumor: 81.8% sens., 100% spec., 91.3% concordance In MPE cell blocks: 40% sens., 100% spec., 72.7% concordance IHC compared to ARMS: In tumor: 54.8% sens., 97.1% spec., 77.3% concordance In MPE cell blocks: 50% sens., 100% spec., 76.9% concordance

**Table 2c:** Results from selected mutational analysis studies

Reference	Cancer Type	Tissue Type	Methods	Genes/ Mutations	Results
[106]	NSCLC	Plasma cfDNA and tumor tissue	NGS deep sequencing	*EGFR* exon 19 del, T790M, L858R	NGS of plasma cfDNA compared to tissue samples: For exon 19 deletions: 50.9% sens., 98.0% spec. For L858R: 51.9% sens., 94.1% spec. For T790M in patients after EGFR-TKI: 94.2% spec.
[107]	NSCLC	Plasma cfDNA, tumor tissue, whole blood circulating tumor cells (CTC)	Targeted NGS with PCR amplification and Cobas® EGFR PCR	*EGFR* T790M	Targeted NGS of CTC samples: T790M detected in 50% of samples; 57% concordance between CTC samples and concurrent tissue samples (74% concordance between CTC samples and all tissue samples) Cobas® EGFR PCR for cfDNA samples: T790M detected in 50% of cfDNA samples; 60% concordance between cfDNA and concurrent tissue samples (61% concordance between cfDNA and all tissue samples) cfDNA samples compared to CTC samples: 65% concordance
[110]	NSCLC	Plasma cfDNA and tumor tissue	Cobas® EGFR PCR and ultra deep NGS	*EGFR* exon 19 del, T790M, L858R	Cobas® PCR for cfDNA: 72% sens., 100% spec.; 71% baseline concordance rate between plasma cfDNA and tissue, 73% progression concordance rate between plasma cfDNA and tissue Ultra Deep NGS for cfDNA: 74% sens., 100% spec.; 74% baseline concordance rate between plasma cfDNA and tissue, 73% progression concordance rate between plasma cfDNA and tissue
[121]	Several tumor types, including NSCLC	Tumor tissue	NGS, IHC, and qualitative Real-Time PCR (qRT-PCR)	*MET*	NGS: *MET* exon 14 mutations detected in 28/933 NSCLC patients (3.0%); 61% deletions, 39% point mutations; 29% of patients also had *EGFR* copy gain; 71% of patients had at least 1 mutation in *TP53* or *MDM2* IHC: c-MET expressed more in stage IV samples with *MET* exon 14 mutations than stage I-III samples with *MET* exon 14 mutations qRT-PCR: *MET* exon 14 skipping occurred in 96% of samples tested
[122, 123]	Several tumor types, including NSCLC	Tumor tissue	Targeted NGS	*MET*	NGS: *MET* mutations detected in 221/38,028 specimens, 3% of which (131) were lung adenocarcinomas; *MDM2* and *CDK4* amplification often occurred with *MET* exon 14 splicing mutations but not with *MET* amplification; Patients with *MET* exon 14 alterations, including c.2888-5_2944del62, c.3028G>C, and c.3028+1G>T, showed partial responses to MET inhibitors
[124] case report	NSCLC- Sarcomatoid	Metastasis of primary lung tumor	NGS	*MET*	Mutations detected: c.2888-5_2890TTAAGATC>A and c.3028+2T>G (both thought to contribute to exon 14 skipping), p.H1094Y (c.3280C>T) Crizotinib response: partial response, decrease in lung mass
[125] case report	NSCLC- Adenocarcinoma	Adrenal lesion- metastasis of primary lung tumor	NGS	*MET*	Mutation detected: Intronic deletion c. 2887-18_2887-7del12 Crizotinib response: lung mass improvement and decrease in size of adrenal lesion after 5 weeks
[126] case report	NSCLC- Adenocarcinoma	Metastasis of primary lung tumor	NGS	*MET*	Mutations Detected: *MET* c.2888-19>2888-2delCTTTCTCTCTGTTTTAA, c.3028G>C, c.3028+1G>A, c.3024_3028delAGAAGGTATATT, p.V1001_F1007del (c.3001_3021delGTAGACTACCGAGCTACTTTT), c. 3028+1G>T, c.3028G>T, and c.3017_3028delCTTTTCCAGAAGGT *MET* TKI response: 3/4 patients who received TKIs exhibited complete or partial responses

### *EGFR* mutations

The epidermal growth factor receptor (EGFR) is a receptor tyrosine kinase responsible for activating downstream proteins and signal cascades, including the RAS/RAF/MAPK and PI3K/AKT pathways [83]. Mutations in *EGFR* result in abnormal receptor activity leading to increased signaling. These mutations are observed in 10% to 30% of NSCLC cases with higher frequencies in the East Asian population than the Caucasian population [83, 84]. In patients with *EGFR* mutations, *EGFR* tyrosine kinase inhibitors (*EGFR* TKIs), including erlotinib and gefitinib, are generally given as first-line treatments [85–87]. However, some patients develop resistance to TKIs, often related to a mutation in exon 20 of *EGFR* resulting in a substitution of methionine to threonine at amino acid position 790 [88]. In personalized medicine, detecting *EGFR*-activating mutations and monitoring for resistance mutations enable physicians to prescribe treatments and modify them as necessary. In order to do this, non-invasive and efficient methods to detect *EGFR* mutations have been developed using cfDNA as a liquid biopsy sample and NGS as technique for mutational analysis.

First, cfDNA samples can be analyzed to detect and monitor *EGFR*-activating mutations in patients with lung cancer. Pasquale et al. used real-time PCR assays to detect *EGF*R L858R mutations and exon 19 deletions in plasma cfDNA from NSCLC patients. They demonstrated that the Plasma-Therascreen^®^ method (ARMS (Amplification Refractory Mutation System) allele-specific real-time PCR using the fluorescent Scorpions probes) and the Peptide Nucleic Acid (PNA)-clamp approach (inhibition of the amplification of the wild-type allele) have similar sensitivities (65.4% and 61.5%, respectively), specificities (100% for both methods), and concordance rates (90.6% and 89.6%, respectively) for the detection of the same *EGFR* mutation present in cfDNA and the corresponding primary tumor [89]. In a different study, Karachaliou et al. analyzed *EGFR* mutations in cfDNA from blood samples collected from 97 untreated patients enrolled in the European Tarceva *versus* Chemotherapy (EURTAC) trial [86, 90]. The authors demonstrated that in patients with the L858R mutation in tissue, who received erlotinib or standard chemotherapy, the detection of L858R in cfDNA is a negative prognostic factor for overall survival (OS), and patients with the L858R mutation in cfDNA had shorter OS than patients with exon 19 deletions [90]. *EGFR* mutation detection in cfDNA was successfully achieved using a PNA-mediated 5′ nuclease real-time PCR assay with 78% sensitivity and 100% specificity [90]. These findings demonstrate the efficacy of this assay in the detection of *EGFR* mutations in cfDNA and shed light on the need for specific combination therapies for patients bearing the L858R mutation in their blood.

Similarly, using Therascreen^®^ EGFR Mutation Test, Goto et al. analyzed *EGFR* mutations in 91 tumor samples and 194 cfDNA samples isolated from patients enrolled in the Iressa Pan-Asia (IPASS) study comparing the efficacy of gefitinib and carboplatin/paclitaxel in patients with lung adenocarcinoma [91, 92]. Among the 86 patients who had mutational data for both tissue and cfDNA, the positive predictive value of the cfDNA *EGFR* mutation test was 100% [91]. However, there was a high false negative rate (56.9%) and a concordance rate of only 66.3%, reflecting either a poor sensitivity of the assay or the absence of cfDNA in some plasma samples [91]. Interestingly, the presence of the mutation in cfDNA or in the tissue permitted the same interpretation of treatment effect (higher progression-free survival (PFS) and higher objective response rate (ORR) with gefitinib *versus* chemotherapy). When compared to the Pasquale el al. study that used the same ARMS-Scorpions PCR approach [89], the sensitivity of the *EGFR* mutation detection test in cfDNA in the Goto et al. study was considerably lower (43%) [91]. The different sample types in each study (plasma or serum) and the modifications made in the assay protocol in Goto's study may explain, at least in part, these discrepancies.

In another study, in which ARMS-Scorpions PCR was also used, a very low concordance rate was observed between *EGFR* mutations found in tumor samples and in plasma cfDNA (17.2%); however, this concordance rate increased to 33.3% in later-stage patients and increased to 36.8% in patients with poorly differentiated tumors [93]. The differences between these results are likely due not only to technical variations but also to differences in staging. Guo et al. [93] used samples from patients across all stages, while Pasquale et al. [89] and Karachaliou et al. [90] used samples from advanced NSCLC patients. Taken together, these studies indicate that cfDNA could be used to detect *EGFR* mutations in patients with late-stage lung cancer using PCR-based assays. Additionally, using a droplet digital PCR (ddPCR) technique, Zhu et al. demonstrated the high sensitivity of this method in detecting *EGFR*-activating mutations in cfDNA from patients with late-stage NSCLC [94].

Next, cfDNA samples can be analyzed for mutations in order to predict tumor response to treatment and to determine if resistance mutations have appeared. For example, using ddPCR, Lee et al. compared tumor tissues with known *EGFR* mutations to plasma cfDNA samples from the same patients and followed the mutational status over time. At baseline, the concordance rate between tissue and plasma cfDNA samples was 87.9% for the L858R mutation and 86.2% for exon 19 deletions [95]. Moreover, 40 out of 40 patients who harbored these mutations at baseline showed a decrease in mutant cfDNA after TKI treatment; however, a T790M mutation was detected in 14 patients who progressed after TKI treatment [95]. In a different study, *EGFR* mutations were detected in 36.4% of tumor tissue samples and 34.7% of plasma cfDNA samples [96]. Furthermore, of the 59 patients who received *EGFR-*TKIs, patients with known *EGFR* mutations in tissue and plasma cfDNA had higher ORR than patients who were wild-type for *EGFR* [96]. These findings are consistent with data from other studies using ddPCR [97, 98]. Finally, Sacher et al. demonstrated that plasma ddPCR is a rapid method to detect *EGFR* exon 19 deletion, L858R, and T790M mutations with high sensitivity and specificity [99].

Importantly, in order to select the most appropriate platform for *EGFR* mutation detection, in particular T790M, as part of the development process of the irreversible T790M-potent EGFR-TKI AZD9291, Thress et al. performed a cross-platform comparison using plasma samples from advanced-stage NSCLC patients enrolled in the multicenter AURA study. The authors demonstrated the high and comparable sensitivity and specificity of digital (BioRad ddPCR^TM^ and beads, emulsion, amplification, and magnetics (BEAMing) dPCR) and non-digital (Cobas® EGFR Mutation Test and Therascreen^®^ EGFR Mutation Test) platforms for the detection of *EGFR*-sensitizing mutations in cfDNA. When comparing the Cobas® EGFR Mutation Test and BEAMing dPCR for *EGFR* T790M detection in cfDNA, the authors observed a high sensitivity with both tests (73% and 81%, respectively). These results support the use of both platforms for *EGFR* T790M detection in cfDNA [100]. Interestingly, Oxnard et al. demonstrated that cfDNA T790M analysis is a viable alternative to tumor T790M genotyping when plasma results are T790M-positive since similar outcomes with AZD9291 were reported in patients with T790M-positive plasma or tissue [101]. Although *EGFR* T790M-positive NSCLC tumors are sensitive to the third generation TKIs such as AZD9291, emergence of resistance to these drugs can still occur. One of the resistance mechanisms is the development of the *EGFR* C797S mutation which blocks drug binding on EGFR. Thress et al. successfully used NGS and ddPCR to detect the *EGFR* C797S mutation in plasma cfDNA from advanced *EGFR*-mutant NSCLC patients with acquired resistance to AZD9291 [102]. Based on these studies, cfDNA can be used to predict a patient's response to TKIs through the analysis of *EGFR* mutations over time.

Moreover, in tumor tissue, targeted NGS has been used to detect the T790M mutation in patients who had *EGFR*-activating mutations and were treated with *EGFR*-TKIs. In addition to *EGFR* T790M, NGS revealed several other acquired resistance mutations, including mutations in *TP53, KDR,* and *KIT* [103], and compound *EGFR* mutations (i.e. more than one mutation in the EGFR tyrosine kinase domain) [104]. Interestingly, patients with compound *EGFR* mutations showed significantly lower OS than patients without such mutations (72.8 months *versus* 83.7 months) [104]. Additionally, to determine if *EGFR* mutations could be found in other sample types, Liu et al. compared mutations found in tumor tissue, plasma cfDNA, and DNA in pleural effusion samples from patients with advanced NSCLC using three different techniques: ARMS PCR, Sanger sequencing, and IHC. Results from this study indicate that pleural effusion samples and plasma cfDNA can be used to determine *EGFR* mutational status and that of these three techniques, ARMS PCR is the most suitable [105]. Moreover, Uchida et al. demonstrated that deep sequencing of plasma cfDNA is highly specific for the detection of *EGFR* mutations, including T790M [106].

Given that the detection rate of *EGFR* mutations can differ significantly between tumor and cfDNA samples, there is a need to address what biopsy sample or combinations of samples provide the most reliable information. To answer this question, in the frame of a multi-institutional Stand-Up-To-Cancer collaboration, Sundaresan et al. analyzed the *EGFR* T790M mutation in plasma cfDNA samples, circulating tumor cell (CTC) samples from whole blood, and tissue samples from TKI-treated patients with advanced NSCLC who were known to harbor *EGFR*-activating mutations. [107]. Notably, the authors found that although concordance rates did not exceed 65%, each biopsy type provided complementary information to the others, indicating that in clinical practice, more than one biopsy or sample type could be required for complete analysis [107]. This conclusion is in agreement with the findings of Oxnard et al. who showed that when *EGFR* T790M mutation is not detected in cfDNA, a tumor biopsy is still needed to determine T790M mutation status [101].

In practice, there is no consensus if one test or another is better suited for the detection of *EGFR* mutations in the clinic, particularly when using cfDNA. However, based on the studies mentioned above and on important, multicenter trials in lung cancer such as EURTAC, IPASS and LUX-Lung 3 [86, 92, 108], the allele-specific PCR-based tests have proven efficient and sensitive in the detection of *EGFR* mutations. Accordingly, some molecular PCR tests received the U.S. Food and Drug Administration (FDA) approval as “companion diagnostic” tests for NSCLC patients. These include, among others, the Cobas^®^ EGFR Mutation test (Roche) (based on real-time allele-specific PCR with Taqman probes) and the Therascreen^®^ EGFR RGQ PCR kit (Qiagen) (based on allele-specific PCR with Scorpions probes) to identify *EGFR* mutations. The Cobas^®^ EGFR Mutation test is designed to detect mutations in *EGFR* exon 19 and the L858R mutation in exon 21. It was approved by the FDA in 2013 following its use in the EURTAC study, the trial that led to the approval of erlotinib as first-line therapy for patients with NSCLC whose tumors harbor an *EGFR*-activating mutation [86, 90, 109]. Importantly, in November 2015 the Cobas® EGFR Mutation Test v2 was also approved by the FDA as a companion diagnostic test for NSCLC using FFPE. This new version of the original test enables an expanded coverage of *EGFR* mutations (42 mutations) including exon 19 deletions, L858R substitution (exon 21) and the resistance mutation T790M (exon 20). Notably, this test can be used with either plasma cfDNA or tissue samples, and in June 2016, the FDA approved it as a companion diagnostic test for NSCLC using cfDNA samples. In the multicenter, prospective TRIGGER trial, Marchetti et al. demonstrated that the Cobas^®^ EGFR Mutation Test v2 and NGS display comparable sensitivities and specificities for the detection of *EGFR* mutations in plasma cfDNA and tissue [110]. In addition, the Therascreen^®^ EGFR RGQ PCR kit (Qiagen) covers 29 mutations in the *EGFR* gene, including exon 19 deletions and the L858R and T790M mutations [109]. In 2013, this test was FDA-approved following its use in the LUX-Lung 3 study that showed increased benefits when lung adenocarcinoma patients with *EGFR*-activating mutations were treated with the TKI afatinib as first-line treatment when compared to cisplatin plus pemetrexed [108, 109]. Furthermore, highly specific and sensitive methods have recently been developed for the detection of rare tumor mutations in cfDNA and would be of interest for clinical application. These methodologies include BEAMing dPCR, Safe-Sequencing System (Safe-SeqS) (digital PCR by sequencing), tagged-amplicon deep sequencing (TAM-seq) and the Cancer Personalized Profiling by deep sequencing (CAPP-seq) [12, 70, 111].

In a clinical setting, deciding how to interpret results from the assays presented here is challenging given that each assay has its own level of detection. For example, levels of detection using ddPCR alone can vary from 0.003% [95] to 0.04% [94, 97]. Other PCR-based assays have levels of detection of 0.1% (PNA-clamp) [89] and of 1-2% (in-house allele-specific PCR) [93, 112]. Furthermore, clinically relevant and FDA approved tests such as the Cobas^®^ EGFR Mutation Test and the Therascreen^®^ EGFR RGQ PCR test, have reported sensitivities of 0.02% and 0.05-2%, respectively [89, 110, 112]. Moreover, different concordance rates between tumor tissue and cfDNA can result from differences in extraction and detection methods, the size of cfDNA fragments, the quantity of cfDNA in the sample, and differences in tumor biology [91]. Therefore, when choosing an assay to use in a clinical setting, extraction methods and the desired limit of detection must be taken into consideration and standardized.

### *MET* mutations

The mesenchymal-epithelial transition factor (*MET*) gene codes for a receptor tyrosine kinase responsible for activation of signaling cascades downstream of SRC homology 2 domain-containing phosphatase 2 (SHP2), PI3K, CRK-like protein (CRKL), among others, and is often involved in oncogenesis in many cancer types, including lung tumors [113]. In several cases, *MET* amplification has been associated with resistance to *EGFR*-TKI therapy [114]. More recently, oncogenic splice-site mutations of *MET* at exon 14 have been discovered and were shown to activate c-MET in patients with NSCLC and SCLC [115–118]. In terms of treatment, *MET*-directed anti-cancer therapies are currently under preclinical and clinical trials and include c-MET inhibitors (Tivantinib, Cabozantinib and Foretinib), anti-MET antibodies (Onartuzumab), and anti-HGF (as the hepatocyte growth factor is a MET ligand) antibodies (Rilotumumab and Ficlatuzumab) [119]. Furthermore, crizotinib is a dual *ALK/MET* inhibitor, presently approved for the treatment of NSCLC patients harboring an *EML4-ALK* fusion. Its efficacy has also been reported in some cases of *MET* amplification, suggesting its potential interest in *MET*-targeted therapies [119].

**Figure 3:**
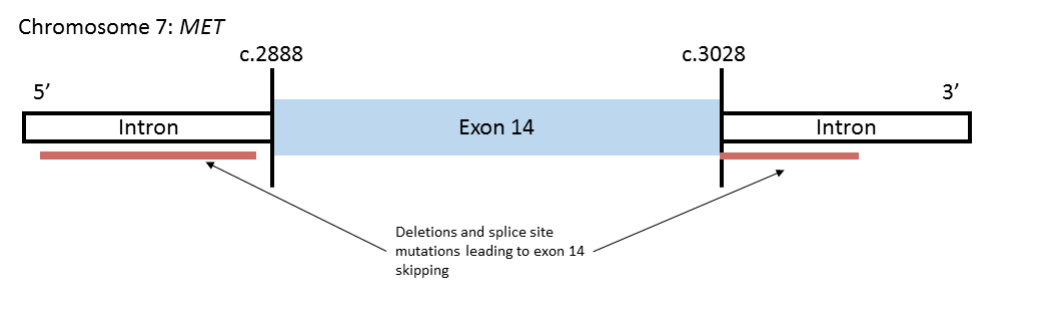
Schematic representation of *MET* mutations that lead to exon 14 skipping Mutations include but are not limited to c.2888-5_2944del62, c.3028G > C, c.3028+1G > T, p.H1094Y (c.3280C > T), c.2888-5_2890TTAAGATC > A, c.2888-19 > 2888-2delCTTTCTCTCTGTTTTAA, c.3028+1G > A, c.3024_3028delAGAAGGTATATT, p.V1001_F1007del (c.3001_3021delGTAGACTACCGAGCTACTTTT), and c.3017_3028delCTTTTCCAGAAGGT.

The availability of these therapeutic options fueled screening of activated MET in a broad range of cancer [120]. NGS has been used to detect *MET* mutations, including exon 14 splice-site mutations that result in exon 14 skipping in patients with lung cancer (Figure 3). In a study to characterize *MET* mutations across many tumor types, *MET* exon 14 mutations were detected in 3% of non-squamous NSCLC cases, mainly adenocarcinomas, were found specifically in older patients and were frequent in early-stage tumors, suggesting their role in lung tumorigenesis [121]. Notably, stage IV NSCLC patients whose tumors exhibited *MET* exon 14 mutations were more likely to display concurrent *MET* amplification and c-MET overexpression [121]. Also, using NGS, Frampton et al. found that *MET* exon 14 splicing alterations, but not *MET* amplification, were often concomitant with the presence of *MDM2/CDK4* amplifications in solid tumor samples [122, 123]. Taken together, these results indicate that *MET* mutations can be found in NSCLC and could aid in diagnosis and in treatment decisions.

Importantly, in two separate case reports, NGS detected the presence of a *MET* exon 14 splice-site mutation in NSCLC tumor tissue, and in each case report, the patient was treated with the *MET* inhibitor crizotinib and showed a partial response or a significant decrease in tumor size [124, 125]. In another study, tumor samples from 178 patients with advanced NSCLC were sequenced using hybrid capture-based NGS. *MET* exon 14 splice-site mutations were detected in 8 patients, and of the 4 patients who received *MET* inhibitors, 3 patients exhibited either complete or partial responses [126]. These case reports and studies highlight the importance of *MET* sequencing in a clinical setting and the need for further investigation of *MET* exon 14 splice-site mutations as possible therapeutic targets in lung cancer.

Interestingly, recent data presented at the 2016 European Lung Cancer Conference (ELCC) highlight the promising use of cfDNA for the detection of *MET* exon 14 mutations. Indeed, the digital sequencing-based liquid biopsy-dedicated Guardant 360 test (Guardant Health) has been used for genomic profiling of cfDNA collected from 54 patients with late-stage or recurrent lung adenocarcinoma. This test detects different alterations in 70 cancer-related genes, including *MET* exon 14 skipping. The data showed an overall concordance rate of 48% between molecular analyses performed in cfDNA and in tissue, with a higher concordance for *EGFR* mutations (71%) (Santos et al., poster presented at 2016 European Lung Cancer Conference). Such studies help pave the way for successful detection of *MET* exon 14 skipping in liquid biopsies.

Based on recent studies, about 3 to 5% of NSCLC patients are expected to harbor *MET* exon 14 skipping, driving cancer progression and metastasis [53, 121, 123, 127]. In the future, NGS will play an essential role in establishing *MET* testing for personalized medicine by identifying patients who have *MET*-driven NSCLC and will benefit from *MET* inhibitors. More importantly, plasma cfDNA, which has shown promising results to detect *MET* exon 14 splice-site mutations, can be evaluated further to monitor the molecular changes upon therapeutic treatment.

## CONCLUSIONS

In the past, lung cancer diagnosis was time-consuming, costly, and invasive as it involved computer tomography (CT), positron emission tomography (PET), and CT-guided needle biopsies. Moreover, treatment could not meet the specific needs of the individual. With the advent of advanced molecular technologies and the knowledge gained from large-scale sequencing projects, there has been a push for individualized diagnosis and treatment in personalized medicine. By using cfDNA for liquid biopsies and NGS as a platform to analyze mutations found in cfDNA, lung cancer diagnosis can become less invasive and less expensive. Furthermore, treatment can be modified to meet a patient's specific needs, and changes in tumors can be monitored over time. To move forward, several technical improvements and standardization steps need to be considered in order to expedite the use of cell-free DNA and NGS in routine clinical practice.
